# PUBLIC POLICY ISSUES OF SIGNIFICANCE IN SOCIAL RESEARCH, POLICY, AND PRACTICE

**DOI:** 10.1093/geroni/igz038.1455

**Published:** 2019-11-08

**Authors:** Robert Harootyan

**Affiliations:** Senior Service America Inc., Silver Spring, Maryland, United States

## Abstract

This presentation will cover public policy issues of significance to the aging population, focusing on the perspective of the social research, policy and practice community and on policies that may improve physical and mental health.

